# New Insights into Biology, Prognostic Factors, and Current Therapeutic Strategies in Chronic Lymphocytic Leukemia

**DOI:** 10.1155/2013/740615

**Published:** 2013-08-22

**Authors:** Piotr Smolewski, Magdalena Witkowska, Anna Korycka-Wołowiec

**Affiliations:** ^1^Department of Experimental Hematology, Medical University of Lodz, Ciolkowskiego 2 Street, 93-510 Lodz, Poland; ^2^Department of Hematology, Medical University of Lodz and Copernicus Memorial Hospital, Ciolkowskiego 2 Street, 93-510 Lodz, Poland

## Abstract

Chronic lymphocytic leukemia (CLL) is characterized by the clonal proliferation and accumulation of mature B lymphocytes. CLL cells show an antiapoptotic profile, suggesting the important role of apoptosis inhibition in the disease development. However, there is some population of proliferating CLL cells, which may also play a role in progression of the disease. There are several newer, biological prognostic factors in CLL. Currently, cytogenetic abnormalities with different prognostic values seem to be the most biologically relevant. During the last decades, the treatment of CLL has been significantly changed. Different strategies such as monotherapy with chlorambucil and purine nucleoside analogues (PNA) used alone or in combination with cyclophosphamide have been introduced. Most recently, immunochemotherapy with anti-CD20 monoclonal antibody, rituximab, combined with fludarabine and cyclophosphamide, became a gold standard of first-line treatment in eligible CLL patients. Currently, new treatment strategies including new monoclonal antibodies, bendamustine, lenalidomide, or inhibitors of several cell signaling pathways are under clinical studies in resistant/relapsed CLL patients. Moreover, allogeneic stem cell transplantation has to be considered, especially in younger high risk patients, for example, those who are resistant to PNA or those with 17p deletion. In this paper, we present the most important recent advances in CLL biology and treatment.

## 1. Introduction

Chronic lymphocytic leukemia (CLL), the most common lymphoid malignancy in the Western world, is characterized by clonal proliferation and accumulation of mature B lymphocytes. CLL is a disease of the adults, with median age at diagnosis ranging between 67 and 72 years [1]. Recent data indicate that approximately 6% of the normal elderly population develops a monoclonal B-cell lymphocytosis (MBL), which can transform into CLL in 1%-2% of cases. Cytogenetically and immunophenotypically MBL cells correspond to leukemic cells in CLL. Typical immunophenotype of the CLL cell is the presence of the B-cell surface antigens CD19, CD22, and CD23 with coexpression of T-cell lineage antigen, CD5, and expression of CD20 and CD79b lower than that observed in normal B cells [2, 3].

Recently, a significant progress has been made in elucidating the biology and improving treatment for CLL. This progress led to the identification of a subset of CLL patient with an early relapse of the disease and high risk of shorter survival, who may potentially benefit from a more aggressive upfront therapy.

## 2. Biology and Pathogenesis of CLL

The development of cytogenetic and molecular biology in the last few years has led to the important progress on the field of CLL studies. It is currently known that CLL cells show an antiapoptotic profile, with strong expression of Bcl-2 protein, which suggests that inhibition of apoptosis is responsible for CLL development. On the other hand, there is some proliferating population of CLL cells. The disease probably develops as a result of accumulation of transformed B cells. In CLL cells, a great imbalance between cell birth and death rate is observed [3, 4]. 

The gene expression profile suggests that CLL cells originate from transformed, antigen-stimulated B cells. These cells known as CD27-positive cell population include memory B cells as well as marginal zone B cells. In CLL cells, several mutated *IGHV* genes are expressed more frequently than in normal B lymphocytes. All those cells express restricted sets of  B-cell receptors (BCR). Unique stereotypy of BCR suggests that antigens play a critical role in CLL pathogenesis. Some of them have already been identified as autoantigens derived from apoptotic cells; epitopes typical for microbial antigens were also reported [3, 4]. 

## 3. Prognostic Factors in CLL

The clinical course and outcome vary among CLL patients. Therefore, there is a need to establish a solid prognostic factors for this disease [5]. CLL patients are currently categorized into risk groups based on the clinical staging systems developed by Rai et al. [6] and Binet et al. in the early 1980s [7]. These classifications are still helpful for dividing patients in regard to the expected overall survival (OS). However, both systems fail to indicate the higher risk of progression among patients in early stages of the disease. These clinical staging systems were complemented by prognostic markers based on peripheral blood or bone marrow examination, such as an identification of atypical morphology of CLL cells, high rate of prolymphocytes, or diffuse infiltration of bone marrow, which are associated with worse outcome [5–8].

Among newer prognostic factors in CLL, there are lymphocyte doubling time (LDT), serum markers, biological prognostic factors (*IGHV*) mutational status, ZAP-70, CD38 expression, and cytogenetic abnormalities [8–10]. LDT longer than 12 months correlates with increased progression-free survival (PFS) and OS. An increase in the lymphocyte count of more than 50% in two months or LDT during less than 6 months is a recommended criterion of active disease and indication for treatment. The latter indication is relevant for patients with the baseline lymphocyte counts more than 30 × 10^9^/L only [11]. 

Serum prognostic factors, such as *β*2-microglobulin (*β*-2M), soluble CD23 (sCD23), or serum thymidine kinase (TK), were indicated as an important prognostic factor for CLL patients. They are relevant markers of proliferative activity and a risk disease progression, correlating with other biological prognostic factors [5]. 

Recently, several biological prognostic markers, including immunoglobulin heavy chain variable region (*IGHV*) mutation status, *TP53* mutations and some cytogenetic abnormalities, cell membrane expression of CD38, and intracellular expression of zeta-associated protein-70 (ZAP-70), have become important prognostic factors [5, 12, 13]. As was mentioned above, CLL patients may have either somatically mutated or unmutated *IGHV* gene, which correlates with a favourable or unfavourable prognosis. Patients with unmutated *IGHV* gene have significantly shorter OS (approximately 8 years) than those with mutated *IGHV* gene (approximately 25 years). The expression of ZAP-70 remains constant over the course of the disease. The cutoff to classify patients as ZAP-70 positive (negative prognostic factor, correlating with unmutated *IGHV* status) or ZAP-70 negative, as measured by flow cytometry, is widely proposed at 20% threshold. However, standardization of ZAP-70 estimation still remains a challenge. Similar correlation with the outcome has been shown for CD38 expression. CD38-positive patients (the threshold >30% CD38+ CLL cells is proposed) were reported to have significantly worse prognosis regarding PFS and OS than those who were CD38 negative. It was observed that CD38 expression on CLL cells correlates with the absence of *IGHV* mutations [3, 5, 8, 12, 13].

Nowadays, the crucial prognostic importance is assigned to chromosomal aberrations in CLL patients. In approximately 80% of patients, there is a mutation detected by fluorescence hybridization in situ (FISH). The most important prognostic role plays deletion in the long arm of chromosome 13 [del(13)(q14)], found in up to 50% cases and connected with a good prognosis, deletion in the long arms of chromosome 11 [del(11)(q22-23)], trisomy 12 (+12), deletion in the short arm of 17 [del(17)(p13)], associated with rapid disease progression, short OS, and resistance to treatment, and deletion of the long arm of chromosome 6 [del(6q)] [5, 10, 13]. 

Deletions of 11q22-q23 and 17p13, resulting in abnormalities of *ATM* and *TP53* genes, respectively, are independent prognostic factors identifying patients with a rapid disease progression and a short OS in a multivariate analysis. Patients with del(11q) have more frequently B symptoms, advanced clinical stage, and extensive peripheral, abdominal, and mediastinal lymphadenopathy. Deletions of 17p and 11q are more often detected in advanced stages of the disease, among patients with unmutated *IGHV* gene. In contrast, deletion of chromosome band 13q14 is associated with a favourable CLL outcome. Moreover, patients with trisomy 12 have a shorter OS than those with 13q deletion [3–5, 8, 10, 13]. 

Taken together, five prognostic categories can be defined based on the presence of cytogenetic abnormalities, which are actually the most relevant for risk estimation in CLL. The prognosis is the worst in patients with del(17p), followed by del(11q), trisomy 12, and normal diploid karyotype, while patients with del(13q) as a single abnormality had the best prognosis. The median OS in these groups ranges from 32 to 133 months and median treatment-free survival ranges between 9 and 92 months [10]. 

Interestingly, deletion of 13q14 may have a role in CLL pathogenesis. Namely, this region encodes a tumor suppressor locus comprising a microRNA (mRNA) cluster, including the miR-15a/16-1 locus, embedded in a long sterile RNA gene. Its deletion leads to development of malignant lymphoproliferative disorders. 

## 4. Treatment of CLL

### 4.1. Standard Chemotherapy and Immunochemotherapy

Despite the great improvement in CLL treatment during the last decades, the disease still remains incurable. Chemotherapy is not routinely indicated in early or stable disease patients. In this group, a “watch-and-wait” approach is widely recommended. Treatment should be introduced in patients with advanced, symptomatic, or progressive disease. The choice of therapy depends on clinical stage, the disease activity, age, and existing comorbidities. 

For many years, chlorambucil (with or without steroids) was the drug of choice in previously untreated patients with progressive or advanced CLL [14]. Although this is no longer a gold standard, this drug is still useful in the treatment of elderly patients with comorbidities (Table 1).

In biologically fit CLL patients, who require prompt treatment, the purine-nucleoside-analogue- (PNA-) based regimens are currently the standard of care. PNAs such as fludarabine (FA), cladribine (2-CdA, 2-chlorodeoxyadenosine), or pentostatin (DCF, 2′-deoxycoformycin) showed high antileukemic activity. Significantly higher overall response (OR), complete remission (CR) rates, and a longer PFS in patients with CLL treated with FA or 2-CdA were documented in several randomized trials [15, 16]. Next, combination of FA or 2-CdA with cyclophosphamide (FC or CC regimens, resp.) appeared to be more effective than PNAs in monotherapy in regard to OR, CR, and PFS [17–19]. 

Monoclonal antibodies (MoAbs), such as anti-CD20 MoAb and rituximab (RIT), showed promising antitumor activity in hematologic malignancies. The results of recent studies showed that RIT in combination with a PNA or PNA and cyclophosphamide can increase the OR and CR rates and PFS time [20]. The combination of RIT with FC (R-FC regimen) demonstrated particularly high rates of OR, CR, and duration of PFS in both previously untreated and relapsed/refractory CLL [25]. Moreover, the followup of the phase III CLL8 trial conducted by German CLL Study Group (GSCG) (R-FC versus FC) showed for the first time that treatment with R-FC can improve OS of previously untreated CLL patients (Table 1) [20]. 

However, R-FC is acceptable for younger, physically fit patients. This regimen has limitations in the unfit group, mainly due to the risk of myelosuppression and other side effects. On the other hand, immunotherapy with R-FC was shown to be highly effective in refractory/relapsed CLL patients (R-FC versus FC; the REACH study) [26]. PFS was prolonged significantly in the R-FC, compared to FC arm (30.6 versus 20.6 months, resp.; *P* = 0.0002). Moreover, OS rates were higher for R-FC than for FC (median 70% versus 58%, *P* = 0.0034), due to superior CR rates (24% versus 13%; *P* = 0.0007) (Table 2).

Recent clinical trials proved that combination of  RIT with pentostatin or 2-CdA and cyclophosphamide (P-CR or R-CC regimens) is a highly active treatment modality in CLL as well [21]. More recently, bendamustine, a bifunctional agent composed of an alkylating nitrogen mustard group and a purine-like benzimidazole ring, has been included in CLL treatment regiments for CLL treatment. In a randomized trial compared activity of bendamustine or chlorambucil in untreated CLL patients [22], OR and CR rates were significantly higher in patients treated with bendamustine. Moreover, the median duration of response was longer after treatment with bendamustine than after chlorambucil (21.8 versus 8.0 months, resp.; *P* < 0.0001) (Table 1).

Currently, bendamustine is considered for CLL treatment either in monotherapy or in combination with RIT. However, relapsed and refractory CLL patients can also benefit from this kind of therapy the most. In such patients, high-dose methylprednisolone (HDMP) alone or in combination with RIT can also be efficacious [23]. Results of several studies showed that HDMP may be useful for resistant patients, including those resistant to FA and/or with *P53* gene abnormalities [27].

More recently, a second-generation, fully human, anti-CD20 antibody, ofatumumab (HuMax-CD20), [34] was found to be effective in the phase III randomized study, as monotherapy for heavily pretreated patients with CLL resistant to F or FA treatment [28]. 

Another MoAb highly active in CLL, alemtuzumab (ALT), is recombinant, humanized anti-CD52 MoAb. In previously untreated patients, an OR rate of more than 80% was achieved, being effective in patients with 17p deletion [24], and in those patients ALT can be used as a first-line treatment (FDA approval in 2007). However, in patients without 17p deletion, especially for those with hyperleukocytosis and no bulky nodal disease, it is recommended as a second- or third-line treatment alone [29] or in combination with other anti-neoplastic drugs (Table 2) [30, 31]. 

### 4.2. Stem Cell Transplantation

Currently, allogeneic hematopoietic stem cell transplantation (alloHSCT) represents the only potentially curative option for CLL, but fully ablative regimens are associated with significant morbidity and mortality. More recently, reduced-intensity conditioning was introduced for alloHSCT (mini alloHSCT). This procedure is better tolerated than the myeloablative one and may be proposed for several patients ineligible for such treatment.

The exact role of alloHSCT in the standard management of CLL patients is still undefined. According to the recent recommendations of the international expert panel [35], indications for alloHSCT include younger patients, requiring treatment with primary FA resistant or in early relapse and with *TP53* gene abnormalities. However, introduction of new targeted therapies with less morbidity, producing durable responses, may redefine the indications for transplantation in CLL [36].

### 4.3. New Anticancer Agents in CLL

Recently, several new agents may be promising in targeting CLL. Novel therapies are being evaluated in preclinical studies and in early clinical trials. These drugs include new MoAbs or agents targeting the antiapoptotic protein Bcl-2 family, receptors involved in mediating survival intracellular signals, antisense oligonucleotides, and antiangiogenic drugs.

GA101 (obinutuzumab) is the first humanized and glycoengineered type 2 anti-CD20 MoAb which enters preclinical and clinical trials. In preclinical studies, GA101 showed increased antibody-dependent cell-mediated cytotoxicity (ADCC) and effectively triggered apoptosis [37]. Currently, the CLL11 trial randomly assigned to 786 “slow-go” CLL patients divided into three arms: chlorambucil with GA101, chlorambucil plus RIT, and chlorambucil alone is conducted. The primary endpoint was PFS. Obtained encouraging results are important because this randomized study is addressed to elderly patients with comorbidities [38]. 

Oblimersen (OBL) is an antisense oligodeoxyribonucleotide blocking transcription of proapoptotic Bcl-2 protein. In a study by O'Brien et al. [32] relapsed/refractory CLL and OBL combined with FC (OBL-FC regimen) provided a significant survival benefit for patients who achieve CR or PR, and those who have FA-sensitive disease. In the phase III trial, CR rate was significantly greater in OBL-FC versus FC arm (17% versus 7%; *P* = 0.025). Among patients with CR, response duration was significantly longer with OBL-FC than with FC (>36 versus 22 months; *P* = 0.03). Additionally, among patients with CR or PR, a significant 5-year OS benefit was observed with OBL-FC (*P* = 0.038).

Other Bcl-2-targeting agents tested in CLL are BH3-mimetics. Obatoclax mesylate (GX15-070) is a small molecule pan-Bcl-2 family antagonist, BH3 mimetic, which enhances TRAIL-mediated apoptosis of tumor cells. Phase I study in patients with advanced CLL was conducted [39]. The study showed biological activity and modest single-agent activity in heavily pretreated patients with advanced CLL. Currently, the trial assessing obatoclax in combination with FA and RIT is ongoing. 

Navitoclax (ABT-263), another BH3 mimetic, specifically inhibits Bcl-2 and related proteins BCL-x_L_ and BCL-w, potently inducing apoptosis of CLL cells *in vitro*. This agent showed activity with relapsed or refractory CLL in phase I/IIa trial [40]. Nine of 26 patients (35%) achieved PS and seven maintained stable disease for more than 6 months. Median treatment duration was 7 months (range 1 to ≥29 months), and median PFS was 25 months. Activity was observed in patients with fludarabine-refractory disease, bulky adenopathy, and del(17p) CLL. Results of combination trials with navitoclax are awaited.

Another modality of treatment in B-cell-derived malignancies is a selective inhibition of the BCR pathway by PCI-32765, an inhibitor of Bruton tyrosine kinase, which plays a role in BCR signaling. According to interim analysis of a phase Ib trial on 54 patients with CLL, either previously untreated or with relapsed/refractory disease >65 years of age [41], fast nodal response was seen in 39 patients with evaluable nodal disease; 87% achieved a nodal PR, with no further lymph node progression. Among 13 patients from the phase Ia trial (median followup of 8 months), there were one with CR and eight with PR. Of the 25 evaluable patients from the current trial, eight (25%) had a PR and 17 (53%) had a nodal PR with lymphocytosis. The drug was very well tolerated and with no myelosuppression. Further clinical studies are ongoing. 

CAL-101 is an inhibitor of phosphatidylinositol 3-kinase (Pl3K) signaling. In preclinical studies, it was found to induce apoptosis of CLL cells. Protein kinase C and PI3K pathways have an influence on the survival of B cells in CLL. In a clinical trial [42], 26% of pretreated CLL patients achieved PR with 80% of patients showing a greater than 50% reduction in lymphadenopathy. The difference between lymph node response compared with PR was due to increase in lymphocytes level in peripheral blood. This suggests that there is a compartment shift. Moreover, 11/13 patients with 17p deletion had a greater than 50% reduction in adenopathy. A trial with combination of CAL-101 and RIT in previously untreated patients in age >65 years is currently enrolling patients.

An interesting type of  new antitumor agents is the small modular immunopharmaceuticals (SMIPs). SMIPs contain variable regions derived from specific antibodies and engineered constant regions encoding human immunoglobulin G1 domains. TRU-016 is humanized anti-CD37 SMIP protein. CLL appears to be a good target for CD37-based therapy because the expression of CD37 is relatively high. TRU-016 effectively mediates ADCC but not complement-dependent cytoxicity (CDC) and triggers CLL cell apoptosis, with synergistic or additive effect with multiple agents in preclinical models. The phase I study administered TRU-016 showed the OR rate of 44% (7/16 patients), with the median reduction in lymphocytosis of 92% [43]. No responses were seen in heavily pretreated patients who had received three or more prior therapies, although there was a significant reduction in lymphocytosis in 61% of patients. Combination clinical trials with TRU-016 have already been initiated.

Alvocidib (flavopiridol, EFC6663) is a synthetic flavone, a potent inhibitor of cyclin dependent kinases (CDKs) 1, 2, and 9. The agent triggers tumor cell p53-independent apoptosis. In a multicenter phase II study on 64 FA-refractory CLL patients, the OR rate was 53%, with 47% PR. The median duration of response was 10 to 12 months. The OR rate in patients with deletions of 17p was 57% [44]. However, toxicity of flavopiridol was significant, including tumor lysis, infections, or diarrhea. 

Finally, the immunomodulatory agent lenalidomide, already found to be extremely active in multiple myeloma, shows activity in CLL. Lenalidomide was evaluated as an initial therapy for elderly patients with CLL. Sixty patients with CLL aging over 65 years received treatment with oral lenalidomide, until disease progression [45]. At a median followup of 29 months, 53 patients (88%) are alive and 32 patients (53%) remain on therapy. Estimated two-year progression-free survival is 60%. The OR rate was 65%, including 10% CR, 5% CR with residual cytopenia, and 43% PR, including 7% nodular PR. The drug was well tolerated. Interestingly, in responding patients an increase in serum immunoglobulin levels was noted as compared with baseline levels. Thus, lenalidomide is effective and well-tolerated treatment strategy for elderly, symptomatic patients with CLL [33].

## 5. Conclusion

CLL resulting from an accumulation of CD5/CD19/CD23 positive cells in bone marrow and peripheral blood is a heterogeneous disease with highly variable clinical course. In some patients, the disease remains stable during the whole lifespan and never needs treatment. In the others, the disease becomes progressive after a variable period of stability and the treatment is necessary. For many years, the standard CLL treatment was based on alkylating agents; however, in the last few decades, several new chemotherapeutics have been synthesized and introduced for clinical practice. Nevertheless, the studies aiming to find out new signaling pathways involved in CLL pathogenesis are needed for identification of novel molecular therapeutic targets which will allow to elaborate the new therapeutic options.

## Figures and Tables

**Table 1:** Larger studies in first-line treatment of CLL patients.

Trial	Regiments	*N*	CR	OR	OS (months/% survivor at time)	Median PFS (months)
Dighiero et al. [14]	Chlorambucil	293	45%	49%	ND	ND
Eichhorst et al. [15]	Chlorambucil	100	0	51%	64	18
	versus fludarabine	93	7%	72%	46	19
Rai et al. [16]	Chlorambucil	181	4%	37%	56	14
	versus fludarabine	170	20%	63%	66	20
Eichhorst et al. [17]	Fludarabine	164	7%	83%	81% at 3 y	20
	versus FC	164	24%	94%	80% at 3 y	48
Robak et al. [18]	CC	192	47%	88%	62.4% at 4 y	28
	versus FC	203	46%	82%	60.6% at 4 y	27
Robak et al. [19]	Cladribine	174	21%	78%	45	27.2
	versus CC	171	29%	83%	48	22.4
	versus CMC	163	36%	80%	46	25.6
Hallek et al. [20]	FC	409	22%	88%	ND	52
	versus rituximab-FC	408	44%	95%	ND	53
Kay et al. [21]	PCR	64	41%	91%	ND	33
Knauf et al. [22]	Chlorambucil	157	2%	31%	ND	8.3
	versus bendamustine	162	31%	63%	ND	21.6
Thornton et al. [23]	HDMP	25	0%	77%	ND	12
Hillmen et al. [24]	Alemtuzumab	149	24%	83%	ND	14.6
	versus chlorambucil	148	2%	55%	ND	11.7

*N*: number of patients in the trial, CR: complete response, PR: partial response, OR: overall response, OS: overall survival, PFS: progression-free survival, ND: no data, FC: fludarabine and cyclophosphamide CC: cladribine and cyclophosphamide, CMC: cladribine, cyclophosphamide, and mitoxantrone, PCR: pentostatin, cyclophosphamide, and rituximab, HDMP: high-dose methylprednisolone.

**Table 2:** Relapsed/refractory immunochemotherapy for CLL patients.

Trial	Regiments	*N*	CR	OR	OS (months)	Median PFS (months)
Robak et al. [25]	RC	18	13%	67%	ND	12
	versus RCC	28	9%	78%	ND	12
Robak et al. [26]	FC	276	13%	58%	46	20.6
	versus RFC	276	24.3%	69.6%	64	30.6
Castro et al. [27]	Rituximab HDMP	14	36%	93%	ND	ND
Wierda [28]	Ofatumumab, FA-ref,	95	0%	51%	14.2	5.5
	versus ofatumumab, BF-ref	111	2%	54%	17.4	5.5
Gritti et al. [29]	Alemtuzumab	18	8%	44%	ND	14.6
Zent et al. [30]	PAR	19	32%	74%	23	7
Badoux et al. [31]	CFAR	31	29%	65%	11	7
O'Brien et al. [32]	FC	120	9%	17%	34	6
	versus FC-oblimersen	121	3%	7%	33	9
Chen et al. [33]	Lenalidomide	25	0%	56%	24	24

*N*: number of patients in the trial, CR: complete response, OR: overall response, OS: overall survival, PFS: progression-free survival, ND: no data, FC: fludarabine and cyclophosphamide, RFC: rituximab, fludarabine, and cyclophosphamide, RC: rituximab, cladribine and RCC: rituximab, cladribine, and cyclophosphamide, PAR: pentostatin, alemtuzumab, and rituximab, CFAR: cyclophosphomide, fludarabine, alemtuzumab, and rituximab, HDMP: high-dose methylprednisolone, BF-ref: fludarabine refractory, FA-ref: fludarabine and alemtuzumab refractory.

## Abbreviations

2-CdA: Cladribine
ABT-263: Navitoclax
alloHSCT: Allogeneic hematopoietic stem cell transplantation
ALT: Alemtuzumab
CLL: Chronic lymphocytic leukemia
CR: Complete remission
FA: Fludarabine
GA101: Obinutuzumab
GX15-070: Obatoclax mesylate
IGHV: Immunoglobulin heavy chain variable region
LDT: Lymphocyte doubling time
MBL: Monoclonal B-cell lymphocytosis
MoAb: Monoclonal antibody
OBL: Oblimersen
OR: Overall response
OS: Overall survival
PFS: Progression-free survival
Pl3K: Phosphatidylinositol 3-kinase
PNA: Purine nucleoside analogue
PR: Partial remission
RIT: Rituximab
SMIPs: Immunopharmaceuticals
ZAP-70: Zeta-associated protein-70.
